# Cell-Free DNA Release Kinetics and Fragmentation Reflect Treatment Response and Resistance In Vitro

**DOI:** 10.3390/cells15171561

**Published:** 2026-08-28

**Authors:** Alexandra Bartolomucci, Laura Kienzle, Sarah Tadhg Ferrier, Benjamin Forgie, Thupten Tsering, Kyle Dickinson, Lorenzo Ferri, Jonathan Cools-Lartigue, Julia V. Burnier

**Affiliations:** 1 Cancer Research Program, Research Institute of the McGill University Health Centre, Montreal, QC H4A 3J1, Canada; alexandra.bartolomucci@mail.mcgill.ca (A.B.);; 2 Department of Pathology, McGill University, Montreal, QC H3A 2B4, Canada; 3 Division of Thoracic Surgery, McGill University Health Centre, Montreal, QC H4A 3J1, Canada; 4 Gerald Bronfman Department of Oncology, McGill University, Montreal, QC H4A 3T2, Canada; 5 Goodman Cancer Institute, McGill University, Montreal, QC H3A 1A3, Canada

**Keywords:** ctDNA, cfDNA, biomarker, chemotherapy resistance, treatment response, esophageal adenocarcinoma

## Abstract

Background: Circulating tumor DNA (ctDNA) has emerged as a clinically valuable biomarker for cancer detection, treatment monitoring, and minimal residual disease assessment. Despite its growing clinical utility, the biological mechanisms governing ctDNA release remain incompletely understood. The objective of this study was to investigate how chemotherapy-induced cytotoxicity and chemoresistance influence the kinetics and fragmentation patterns of cfDNA released by cancer cells in vitro. Methods: Human lung adenocarcinoma (A549) and esophageal adenocarcinoma (FLO-1, OE19) cell lines were used to investigate cell-free DNA (cfDNA) release and fragmentation in vitro. Cancer cells were treated with chemotherapy (cisplatin and 5-fluorouracil), and cfDNA released into the culture medium was quantified as total cfDNA by Qubit fluorometry and mutation-specific cfDNA by droplet digital PCR (ddPCR). A cisplatin-resistant OE19 model was generated to directly compare cfDNA release kinetics between chemosensitive and chemoresistant cells. Fragment size distributions were determined and evaluated alongside cell death mechanisms assessed by flow cytometry. Results: cfDNA release correlated positively with viable tumor cell number across all cell lines. Chemotherapy increased per-cell cfDNA release in all cell lines. Chemosensitive OE19 cells exhibited significantly higher cfDNA release compared to chemoresistant OE19 cells following cisplatin exposure, with distinct temporal release kinetics. Additionally, chemotherapy treatment induced a shift toward the release of larger DNA fragments in both chemosensitive and resistant cells. This was accompanied by changes in PI-positive and Annexin V/PI double-positive cell populations, suggesting altered cell death processes following cisplatin treatment. Conclusions: Chemotherapy-induced cytotoxicity significantly influences cfDNA release kinetics and fragmentation patterns, with distinct effects in chemosensitive and chemoresistant cancer cells. These findings provide mechanistic insight into tumor-derived DNA release biology and may have important implications for the interpretation, timing, and standardization of liquid biopsy testing during treatment. Furthermore, they establish in vitro cancer models as a valuable platform for studying cfDNA dynamics and informing preclinical therapeutic development.

## 1. Introduction

Circulating tumor DNA (ctDNA), referring to fragmented DNA shed from tumor cells into the circulation, has emerged as a powerful and minimally invasive biomarker for cancer detection, prognostication, and disease monitoring. Among its most impactful clinical applications are the assessment of treatment response and the detection of minimal residual disease (MRD), where changes in ctDNA levels can provide an early indication of therapeutic efficacy or disease recurrence [1]. As ctDNA increasingly informs longitudinal clinical decision-making, understanding the biological mechanisms governing its release and clearance has become essential for the accurate interpretation of liquid biopsy results and standardization of ctDNA analyses across clinical settings.

All cells produce cell-free DNA (cfDNA), small fragments of DNA released primarily through cell death processes, including apoptosis, necrosis, and pyroptosis, as well as through active secretion [2,3]. ctDNA is a small proportion of total cfDNA in the blood, corresponding only to the fraction specifically from cancer cells. While the mechanisms underlying cfDNA release have been defined, the extent to which different cellular states and external stressors influence ctDNA release from cancer cells remains unclear. Cancer therapies such as chemotherapy can induce varying degrees of cytotoxicity depending on tumor cell sensitivity, but how these differential responses impact ctDNA release quantity and timing has not yet been fully characterized. Certain clinical studies have shown that treatment-induced cytotoxicity may lead to transient increases in ctDNA levels in some patients [4,5,6]. Of particular concern, transient spikes in ctDNA following anti-cancer treatment may be misinterpreted as disease progression rather than reflecting therapy-induced tumor cell death, a potential confound that underscores the need to better understand the biological determinants of ctDNA release.

cfDNA fragmentomic approaches have similarly emerged as powerful additions to liquid biopsy analyses. Fragmentomics refers to the analysis of cfDNA fragmentation patterns, fragment sizes, and end characteristics as disease biomarkers [1,7]. These features are increasingly assessed in clinical studies [8,9,10,11]. Because cfDNA fragmentation patterns are shaped by the biological processes underlying DNA release [7], cytotoxic treatment-induced cell death may alter cfDNA fragmentation profiles, with direct implications for the interpretation and reproducibility of fragmentomics-based assays.

In the present study, we sought to better determine the impact of cytotoxic treatments on both the release kinetics and fragmentation patterns of cfDNA using in vitro cell models. We developed a chemoresistant esophageal adenocarcinoma (EAC) model to directly compare cfDNA release between chemosensitive and resistant cell populations. By evaluating how treatment response influences cfDNA emission and fragmentation, this work provides critical insight into tumor-derived DNA release biology and highlights important considerations for the timing, interpretation, and standardization of ctDNA analyses in the clinic.

## 2. Materials and Methods

### 2.1. Cell Lines and Culture Conditions

The human lung (A549) and esophageal (FLO-1, OE19) cancer cell lines were kindly provided by Dr. Lorenzo Ferri (Research Institute of the McGill University Health Centre, Montreal, QC, Canada). A549 cells were grown in F-12K medium (Wisent Inc., Saint-Jean-Baptiste, QC, Canada) supplemented with 10% fetal bovine serum (FBS; Corning Inc., Corning, NY, USA), 1% penicillin–streptomycin antibiotics, and 2 mM glutagro (Corning Inc.). FLO-1 cells were grown in DMEM (Wisent Inc.) supplemented with 10% FBS, 1% penicillin–streptomycin, and 2 mM glutagro. OE19 cells were grown in RPMI 1640 (Wisent Inc.) supplemented with 10% FBS, 1% penicillin–streptomycin, and 2 mM glutagro. All cell lines tested negative for mycoplasma contamination.

Cisplatin (Sigma-Aldrich, St. Louis, MO, USA, catalog no. P4394) was dissolved in 0.9% NaCl (Saline). 5-Fluorouracil (5-FU, Sigma-Aldrich, catalog no. F6627) was dissolved in dimethyl sulfoxide (DMSO, Corning Inc.). For cfDNA release experiments, cells were treated with 0.002 mg/mL cisplatin or 0.0032 mg/mL 5-FU. These fixed concentrations were selected as cytotoxic treatment conditions that produced measurable treatment effects over the experimental incubation period. In each experiment, equivalent volumes of saline or DMSO were added to control cells. Since we had observed that in the 24 h after trypsinization and cell attachment, cells release large amounts of cfDNA (Supplementary Figure S1), in all experiments herein we allowed cells to attach and grow for 24 h after passaging before introducing experimental conditions. After experimental incubation, cell culture supernatant was collected, spun first at 300× *g* for 5 min, then again at 1000× *g* for 10 min before freezing at −20 °C until cfDNA isolation.

### 2.2. Determining Cell Numbers

Since preliminary experiments suggested that detached cells present in the cell culture supernatant may contribute to cfDNA release (Supplementary Figure S2), detached and attached cells were both counted for normalization of cfDNA levels to total cell number. Briefly, after the cell culture supernatant was collected and spun at 300× *g* for 5 min, the pelleted detached cells were resuspended in 1 mL media. Likewise, attached cells were recovered using 0.05% trypsin/EDTA (Wisent Inc.) and resuspended in 1 mL media. Then, 10 μL of cell suspension was mixed with 10 μL of trypan blue solution (0.4%; Invitrogen, Carlsbad, CA, USA). Total cell numbers were obtained using a Countess 3 Automated Cell Counter (Invitrogen). All samples were read in duplicate. The corresponding attached, detached, and total cell counts, together with unnormalized and cell-number-normalized cfDNA measurements for each biological replicate, are provided in Supplementary Table S1.

### 2.3. DNA Isolation and Quantification

cfDNA was isolated from cell culture supernatant with the QIAamp Circulating Nucleic Acid kit (QIAGEN, Hilden, Germany) using the protocol for extraction of DNA from 3 mL of urine, modified by not adding buffer ATL (tissue lysis buffer, Qiagen), as well as performing a two-step elution. A first elution with 20 μL followed by a second elution with 10 μL buffer AVE (QIAGEN) was performed.

Total cfDNA was quantified using the Qubit fluorometer dsDNA high sensitivity kit (catalog no. Q32854, Invitrogen) following the manufacturer’s protocol. Samples were vortexed and briefly centrifuged before quantification using 1 μL of DNA. All samples were read in duplicate.

To assess potential background DNA contributing to Qubit-based total cfDNA measurements, including DNA potentially originating from FBS-containing culture media, media-only controls and PBS extraction blanks were processed using the same centrifugation, DNA isolation, elution, and Qubit quantification procedures as the experimental samples, as detailed in Supplementary Figure S3.

### 2.4. Droplet Digital PCR (ddPCR) Conditions

Mutation-specific cfDNA was quantified by ddPCR using assays targeting cell line-specific mutations in A549, FLO-1, and OE19 cells. Primers, probes, and synthetic DNA controls (gBlock gene fragments containing the corresponding mutant or wild-type sequence) were designed by and purchased from Integrated DNA Technologies (Coralville, IA, USA). Primer and probe sequences can be found below in Table 1.

Each mutation-specific assay was optimized using an annealing temperature gradient with the corresponding mutant and wild-type gBlock controls. The annealing temperature providing clear separation of mutant-positive and negative droplet populations was selected for each assay and was maintained for all subsequent experiments. Temperature-gradient plots are shown in Supplementary Figure S4.

ddPCRs were performed as per the manufacturer’s protocol, using 10 μL of 2× ddPCR Supermix for probes (No dUTP) (Bio-Rad, Hercules, CA, USA), 900 nM forward/reverse primers (Integrated DNA Technologies), 250 nM FAM/HEX Affinity Plus qPCR probes (Integrated DNA Technologies), 2 μL of DNA, and nuclease-free water (Integrated DNA Technologies). No-template controls (NTC) were added to each assay. For each reaction, the mix was added to the cartridge, followed by 70 μL of Droplet Generation Oil (Bio-Rad). Droplets were generated using the QX200 Droplet Generator (Bio-Rad) and transferred to a 96-well PCR plate (Bio-Rad). PCR reactions were run as follows: 10 min at 95 °C, followed by 50 cycles of 30 s at 95 °C, 1 min at the assay-specific optimized annealing temperature of 54–62 °C (listed in Table 1 and shown in Supplementary Figure S4) and 30 s at 72 °C, and finally, 5 min at 98 °C. A ramp rate of 2 °C/s was used. The plate was read using the QX200 Droplet Reader (Bio-Rad), and data were analyzed using QuantaSoft Analysis Pro software (version 1.7.4.0917). All samples were analyzed in technical duplicate. Samples with <10,000 droplets were excluded from analysis.

Fluorescence thresholds were established separately for each mutation-specific assay based on the droplet distributions of the NTC and the corresponding mutant and wild-type gBlock controls. Thresholds were positioned to distinguish mutant-positive from negative droplet populations and were applied consistently across samples analyzed using the same assay. Representative two-dimensional droplet plots showing wild-type and mutant gBlock controls, NTCs, and experimental samples are provided in Supplementary Figure S5.

Mutant target concentrations were determined using QuantaSoft Analysis Pro software based on Poisson modeling of positive and negative droplet populations. The estimated total number of mutant copies in the extracted cfDNA sample was then calculated as follows: Mutant copies = $(mean\;mutant\;target\;concentration)\times 20\;\mu L\;PCR\;reaction\times \frac{30\;\mu L\;elution\;volume}{2\;\mu L\;DNA\;loaded\;into\;PCR\;reaction}$.

Blank background was evaluated separately for each mutation-specific assay using 10 NTC reactions performed across 10 independent experiments per assay, with a maximum of one mutant-positive droplet observed in any individual NTC reaction. Based on this blank-background assessment, samples with ≤2 mutant-positive droplets were conservatively classified as negative for mutation-specific cfDNA.

### 2.5. Generating Cisplatin-Resistant OE19 Cells

A chemotherapy-resistant OE19 cell line was generated as previously described by Hummel et al. [12]. Briefly, OE19 cells were exposed to 5 μmol/L of cisplatin for a period of 72 h, after which time the media was removed, and the cells were allowed to recover in chemotherapy-free media. Once the cells were 70–80% confluent, they were passaged and incubated for 24 h before repeating this process. This procedure was repeated for 3 months. Chemoresistant phenotypes were maintained through cisplatin treatments every 3 passages. Prior to experimental assays, cisplatin-resistant OE19 cells were cultured for at least one complete passage in cisplatin-free medium.

### 2.6. CCK-8 Assay

Cells were plated in a 96-well plate at 4000 cells per well in 100 μL of medium in triplicate and incubated for 24 h. After 24 h, cisplatin (at concentrations of 64, 32, 16, 8, 4, 2, and 1 µg/mL) or saline of the same volume was added. The cells were incubated for 48 h. After this time, 10 μL of CCK-8 reagent (Dojindo Molecular Technologies Inc., Rockville, MD, USA) was added to the culture medium, and the plate was incubated at 37 °C for 2 h. The absorbance was measured at 450 nm by an Infinite M200Pro microplate reader (Tecan, Männedorf, Switzerland).

### 2.7. DNA Fragment Size Analysis

DNA fragment size was assessed using a 4150 TapeStation automated electrophoresis system (Agilent Technologies, Santa Clara, CA, USA) with Genomic DNA and Cell-free DNA ScreenTape assays to capture complementary portions of the extracellular DNA size distribution. Genomic DNA ScreenTape was used to determine the average DNA fragment size across the 150–50,000 bp range, whereas Cell-free DNA ScreenTape was used to quantify the proportion of short DNA fragments, defined as fragments between 50 and 700 bp. Electropherograms were analyzed using the TapeStation Controller and TapeStation Analysis software (version 5.2). All samples were analyzed in technical duplicate.

### 2.8. Annexin V/Propidium Iodide (PI) Labeling

Cell death was analyzed using Annexin V/PI labeling. Dissociated cells were resuspended in Annexin V binding buffer and stained using the Alexa Fluor 488-Annexin V/Dead Cell Apoptosis Kit (Thermo Fisher Scientific, Waltham, MA, USA) following the manufacturer’s instructions. Data acquisition was performed using the BD FACS-CANTO II system (BD Biosciences, San Jose, CA, USA). Approximately 20,000 cells were acquired per sample at a rate of about 500 cells per second. Data analyses were performed using FlowJo V11 software to determine the fractions of apoptotic or necrotic cells. Unstained controls and single-stained Annexin V and PI controls were included. Fluorescence compensation was performed using appropriate single-stained controls to correct for spectral overlap between the FITC and PE detection channels. Events were then gated by FSC-A/SSC-A to exclude debris, followed by singlet gating, and Annexin V/PI positivity was quantified within the singlet population using quadrant gating. A representative gating strategy can be found in Supplementary Figure S6.

### 2.9. Statistical Analyses

Statistical analyses were performed using the GraphPad Prism 10 software (v.10.6.1). Data are presented as mean ± standard deviation (SD) from independent biological replicates. Linear associations between plated cell number and cfDNA release were assessed using Pearson’s correlation coefficient. Comparisons between two groups, including predefined vehicle-matched treatment comparisons, were performed using two-tailed unpaired *t*-tests. For experiments involving more than two groups or multiple experimental factors, two-way ANOVA was used to assess the effects of experimental factors and their interaction, followed by Sidak’s multiple comparisons test for pairwise comparisons. CCK-8 dose–response curves were fitted by nonlinear regression using a four-parameter variable-slope inhibitory dose–response model. The Top parameter was constrained to 100% normalized cell viability, and the Bottom parameter was constrained to values greater than 0, whereas IC50 and Hill slope were freely fitted. IC50 values are reported with 95% profile-likelihood confidence intervals, and goodness of fit was assessed using R^2^. Differences were considered statistically significant at *p* < 0.05.

## 3. Results

### 3.1. cfDNA Amount Released In Vitro Correlates to Tumor Cell Number

**Figure 1:**
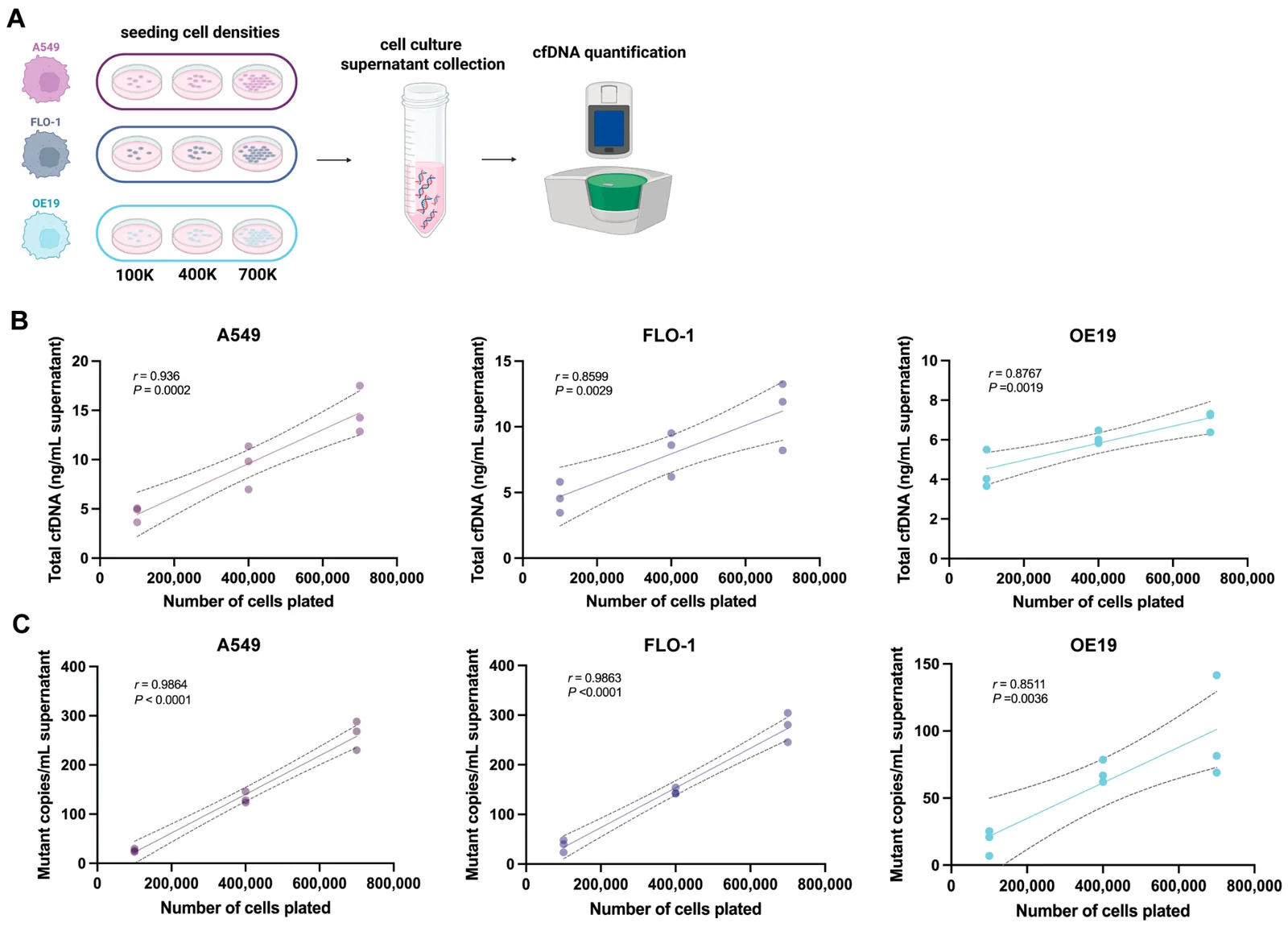
cfDNA amount released in vitro correlates with tumor cell number. (**A**) Experimental design for cell-density cfDNA release assay. A549, FLO-1, and OE19 cells were seeded at increasing cell densities, and conditioned culture medium was collected after 24 h for cfDNA isolation and quantification. Associations between plated cell number and (**B**) total cfDNA measured by Qubit fluorometry and (**C**) mutation-specific cfDNA measured by ddPCR are shown. Individual points represent independent biological replicates (*n* = 3 per cell density). Linear regression lines with 95% confidence bands are shown. Pearson correlation coefficients (*r*) and two-tailed *p*-values are displayed for each cell line.

To determine whether cfDNA release correlates with viable cancer cell number, A549, FLO-1, and OE19 cells were seeded at different densities, followed by cell culture supernatant collection and cfDNA isolation and quantification. The amount of cfDNA released increased with seeding density across all cell lines and quantification methods (Figure 1), with significant positive associations between plated cell number and total cfDNA measured by Qubit fluorometry in A549 (r = 0.9357, *p* = 0.0002), FLO-1 (r = 0.8599, *p* = 0.0029), and OE19 (r = 0.8767, *p* = 0.0019) cells, and between plated cell number and mutation-specific cfDNA measured by ddPCR in A549 (r = 0.9864, *p* < 0.0001), FLO-1 (r = 0.9863, *p* < 0.0001), and OE19 (r = 0.8511, *p* = 0.0036) cells (Pearson correlation analyses). Low but measurable Qubit fluorescence signals were detected in complete-media controls and PBS extraction blanks (Supplementary Figure S3). Because matched experimental conditions used the same complete media and processing workflow, this background was shared across comparisons and is unlikely to explain the observed cell-number-dependent associations, which were also supported by mutation-specific ddPCR.

### 3.2. cfDNA Release from Cancer Cells Increases After Chemotherapy Treatments

**Figure 2:**
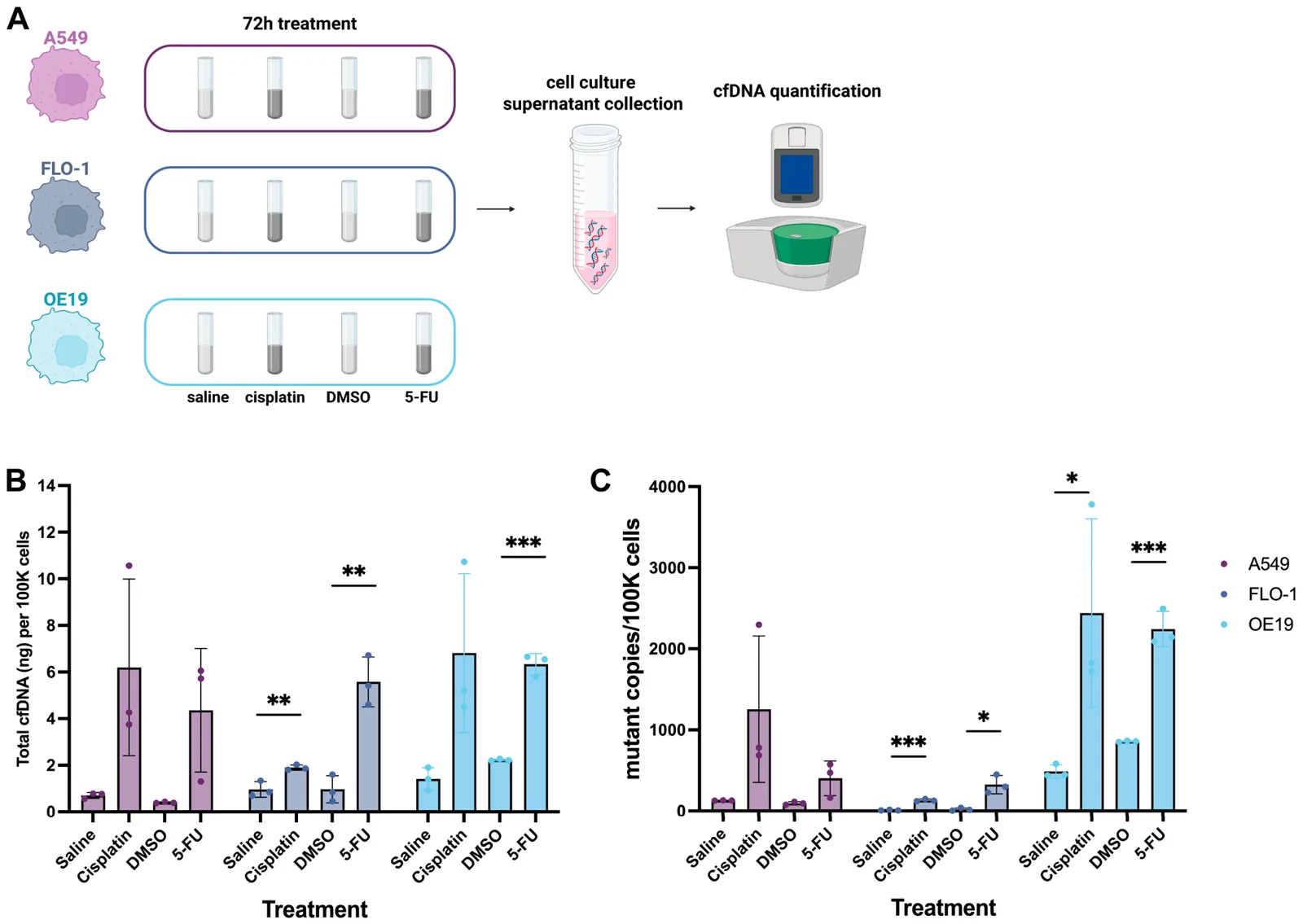
In vitro chemotherapy treatment leads to increased release of cfDNA. (**A**) Experimental design for chemotherapy-induced cfDNA release assay. Cisplatin was dissolved in 0.9% NaCl and compared with a volume-matched saline vehicle control, whereas 5-FU was dissolved in DMSO and compared with a volume-matched DMSO vehicle control. The final vehicle concentrations of saline and DMSO in the culture medium were 0.4% (*v*/*v*) and 0.032% (*v*/*v*), respectively. cfDNA concentrations in conditioned media after 72 h of incubation were measured by (**B**) Qubit Fluorometer for total cfDNA and (**C**) ddPCR assay for mutation-specific cfDNA. All values were normalized to total cell count at the time of supernatant collection. Data represent mean ± SD (*n* = 3 independent biological replicates). Statistical significance was assessed using two-tailed unpaired *t*-tests comparing cisplatin-treated cells with saline controls and 5-FU-treated cells with DMSO controls within each cell line. * *p* < 0.05, ** *p* < 0.01, *** *p* < 0.001.

Next, we wanted to determine the impact of chemotherapy treatments on cfDNA release from the three cell lines. Since chemotherapy treatment altered cell proliferation and total cell abundance over the experimental incubation periods, cfDNA measurements were normalized to total cell number at the time of supernatant collection. This approach was used to assess relative cfDNA release on a per-cell basis. A549, FLO-1, and OE19 cells were seeded at 50,000, 125,000, and 500,000 cells, respectively, so as to reach 80% confluency by the time of cell culture supernatant collection, accounting for differing cell doubling rates [13]. Cells were treated with 0.002 mg/mL of cisplatin and 0.0032 mg/mL of 5-FU. Volume-matched saline and DMSO vehicle controls were used for cisplatin and 5-FU, respectively. Cell culture supernatant was then collected, and cfDNA was isolated and quantified. An increase in per-cell cfDNA release was observed in each cell line following chemotherapy treatment compared to controls (Figure 2). These findings demonstrate that chemotherapy-induced cytotoxicity increases cfDNA release from lung and esophageal cancer cells in vitro.

### 3.3. cfDNA Release Kinetics Differ Between Chemosensitive and Chemoresistant Cells Following Treatments

**Figure 3:**
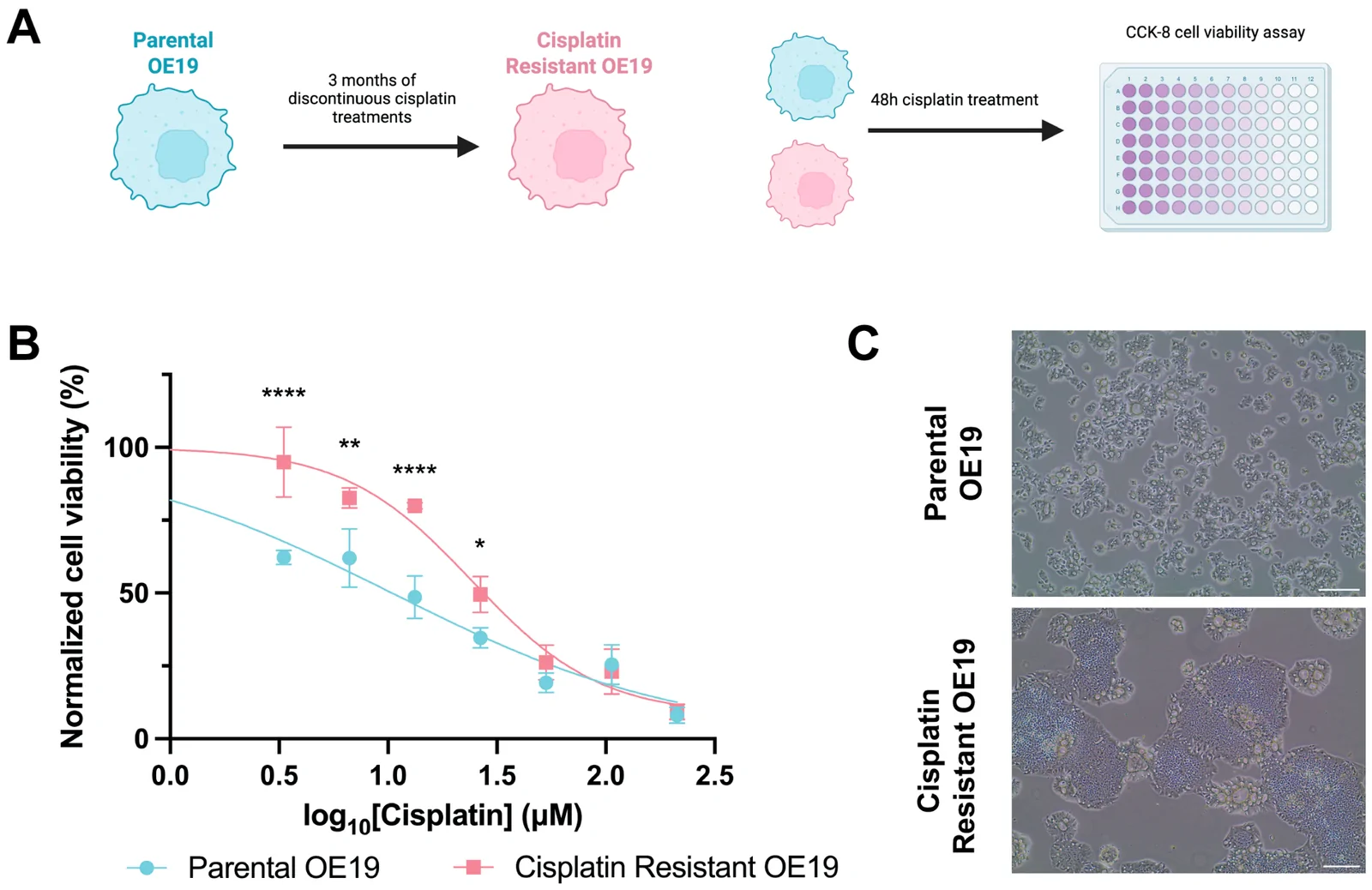
A chemoresistant model of the EAC cell line OE19 was established. (**A**) Schematic of generation and validation of cisplatin-resistant OE19 cells. (**B**) Dose–response curves showing cell viability of parental OE19 and Cisplatin Resistant OE19 cells as measured by CCK-8 assay after treatment with various concentrations of cisplatin for 48 h. Viability was normalized to the matched saline-treated control. Dose–response curves were fitted by nonlinear regression using a four-parameter variable-slope inhibitory dose–response model. The Top parameter was constrained to 100% normalized cell viability, and the Bottom parameter was constrained to values greater than 0, whereas IC50 and Hill slope were freely fitted. Data represent mean ± SD (*n* = 3 independent biological replicates). Two-way ANOVA with Sidak’s multiple-comparisons test revealed significant effects of concentration, cell line, and their interaction (all *p* < 0.0001). Asterisks indicate significant differences between cell lines at individual concentrations: * *p* < 0.05, ** *p* < 0.01, **** *p* < 0.0001. (**C**) Morphology of OE19 and Cisplatin Resistant OE19 cells. Scale bar = 100 µm.

In order to gain a deeper understanding of the underlying mechanisms mediating release kinetics of cfDNA from cells, we wanted to determine how chemoresistance impacts cfDNA release. To do this, we first developed a chemotherapy-resistant model of EAC using OE19 cells treated with three months of discontinuous cisplatin (Figure 3A). To validate the chemoresistant phenotype, parental and cisplatin-resistant OE19 cells were subjected to CCK-8 viability assays across a range of cisplatin concentrations. Cisplatin-resistant OE19 cells exhibited significantly increased cell viability after 48 h of treatment compared to parental cells across several concentrations tested (Figure 3B), producing a right-shifted dose–response curve. Consistent with this, nonlinear regression analysis showed a 2.34-fold increase in the half-maximal inhibitory concentration (IC50) in cisplatin-resistant OE19 cells compared with parental cells (24.40 µM, 95% CI: 19.25–32.76 µM versus 10.43 µM, 95% CI: 6.298–13.17 µM, respectively). The fitted dose–response models showed good agreement with the experimental data (R^2^ = 0.9636 and 0.9471 for cisplatin-resistant and parental OE19 cells, respectively). Additionally, morphological changes were observed following three months of selection, including altered cell–cell adhesion and a more uniform cell morphology, consistent with clonal selection of a resistant subpopulation under sustained drug pressure (Figure 3C).

**Figure 4:**
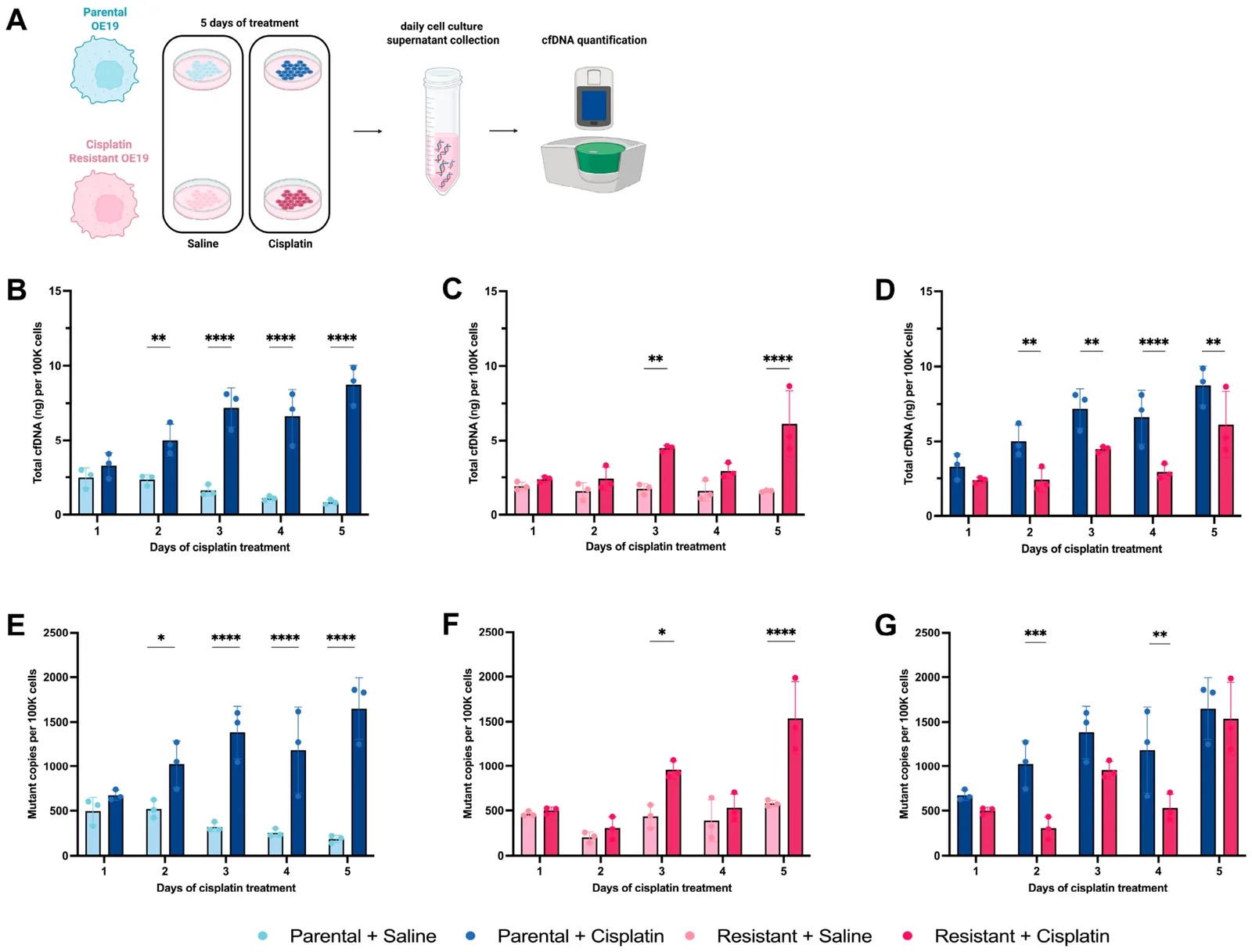
cfDNA release is larger in chemosensitive cells as compared to resistant cells after cisplatin treatments. (**A**) Experimental design for longitudinal analysis of cfDNA release kinetics. Parental (chemosensitive) OE19 cells and cisplatin-resistant OE19 cells were treated with daily cisplatin over five days, and cell culture supernatant was collected at 24-h increments. cfDNA levels were quantified using (**B**–**D**) Qubit fluorometry for total cfDNA and (**E**–**G**) ddPCR for mutation-specific cfDNA. All values were normalized to total cell count at the time of supernatant collection. Across both assays, cfDNA levels increased over time following treatment, with significantly higher cfDNA release observed in chemosensitive OE19 cells compared to resistant cells. Statistical analysis was performed using two-way ANOVA with Sidak’s multiple comparisons test. Significant effects of time, experimental condition, and their interaction were observed (all *p* < 0.0001). Data represent mean ± SD (*n* = 3 independent biological replicates). * *p* < 0.05, ** *p* < 0.01, *** *p* < 0.001, **** *p* < 0.0001.

To directly compare cfDNA release dynamics between chemosensitive and resistant cells, parental and cisplatin-resistant OE19 cells were treated with daily cisplatin over five days, and cell culture supernatant was collected longitudinally at 24-h intervals and quantified (Figure 4A). In both cell populations, per-cell cfDNA levels increased following cisplatin exposure (Figure 4B,C,E,F). However, importantly, chemosensitive OE19 cells consistently released more cfDNA than chemoresistant cells following cisplatin exposure, with significant differences observed at multiple timepoints (Figure 4D,G, Sidak-adjusted *p* < 0.05). Thus, despite continued drug exposure, resistant cells exhibited attenuated cfDNA release. Taken together, these findings demonstrate that per-cell cfDNA release is strongly influenced by cytotoxic treatment and evolves over time. Most critically, release magnitude differs according to chemosensitivity, suggesting that the magnitude of tumor-derived cfDNA release reflects the degree of chemotherapy-induced cytotoxicity.

### 3.4. Cisplatin Treatment Alters Cell Death Profiles and Leads to a Shift in cfDNA Fragment Size

**Figure 5:**
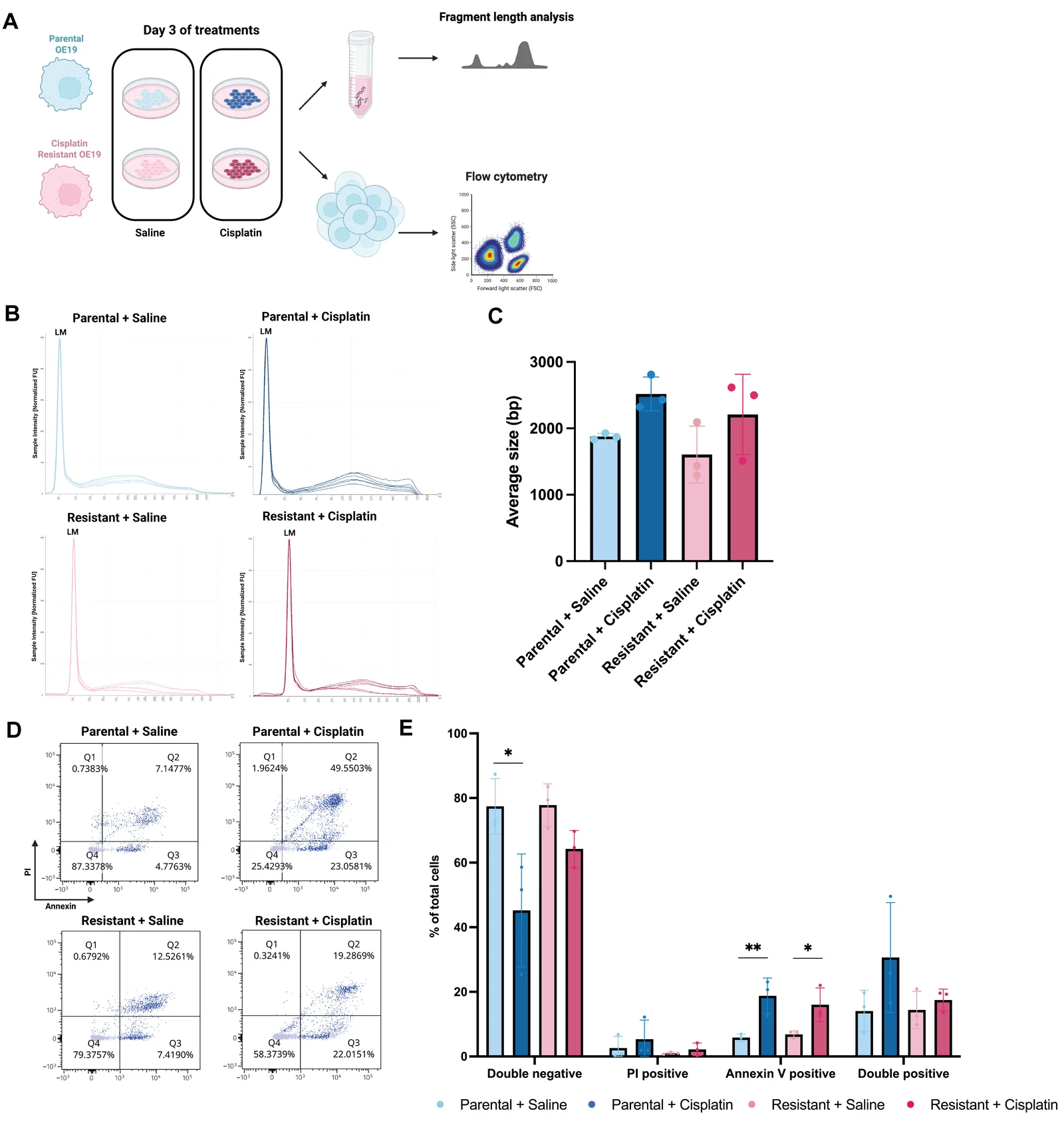
Cisplatin treatment leads to a shift in cfDNA fragment size and altered cell death profiles. (**A**) Experimental design for cell death and DNA fragment size analysis. (**B**) Genomic DNA ScreenTape electropherograms of cfDNA isolated from conditioned medium of parental OE19 and cisplatin-resistant OE19 cells following saline or cisplatin treatment. For each condition, traces from 3 independent biological replicates, each analyzed in 2 technical replicates, are shown (6 traces per condition). The prominent peak at the lower size limit corresponds to the internal TapeStation lower marker (LM). FU, fluorescence units. (**C**) Average cfDNA fragment size measured using the Genomic DNA ScreenTape assay. Data represent mean ± SD (*n* = 3 independent biological replicates). (**D**) Representative Annexin V/propidium iodide (PI) flow cytometry plots of parental OE19 and cisplatin-resistant OE19 cells following 3 days of saline or cisplatin treatment. (**E**) Quantification of cell populations, including double-negative, PI-positive, Annexin V-positive, and double-positive cells. Data represent mean ± SD (*n* = 3 independent biological replicates). Statistical significance was assessed using two-way ANOVA with Sidak’s multiple comparisons test, comparing treatment effects within each cell line. * *p* < 0.05, ** *p* < 0.01.

To investigate the impact of cytotoxic treatment on cfDNA fragmentation, fragment size distributions were assessed using the Agilent TapeStation 4150 automated electrophoresis system. Fragment size profiling was performed on Day 3 of treatment, matching the timepoint used for flow cytometric assessment of cell death (Figure 5A). Within cfDNA isolated from cell culture medium, various fragment size peaks were observed (Figure 5B, Supplementary Figure S7A). Peaks were identified at approximately 167 bp and its multiples, consistent with mono-, di-, tri-, and tetra-nucleosomal fragments generated during apoptosis [14,15] (Supplementary Figure S7A). Small peaks at approximately 50–100 bp were also detected, likely corresponding to linker DNA fragments [14]. Additionally, larger fragments of approximately 1000–15,000 bp were detected, consistent with larger extracellular DNA fragments that have previously been associated with necrotic cell death [16]. Cisplatin treatment produced a consistent shift toward larger extracellular DNA fragments in both parental and cisplatin-resistant OE19 cells. Average fragment size increased following treatment in both cell populations (Figure 5C). Quantitative analysis of predefined fragment size ranges further displayed that the proportion of total cfDNA within the 134–200 bp and 50–700 bp ranges decreased following cisplatin exposure, whereas the proportions within the 1000–15,000 bp and >1000 bp ranges increased (Supplementary Figure S7B). These findings suggest that cytotoxic treatment may alter the mode of cfDNA release, with increased abundance of larger fragments potentially reflecting changes in cell death and extracellular DNA processing.

To further investigate this, we performed Annexin V/PI staining followed by flow cytometry for cells on day 3 of cisplatin treatment. Consistent with the reduced cisplatin sensitivity observed by CCK-8 cell viability assay, cisplatin significantly reduced the viable Annexin V/PI double-negative fraction in parental OE19 cells (Sidak-adjusted *p* = 0.0120), whereas a smaller, non-significant reduction was observed in cisplatin-resistant OE19 cells (Sidak-adjusted *p* = 0.2908) (Figure 5D,E). Cisplatin treatment was also associated with increased PI-positive and Annexin V/PI double-positive cell populations in both cell lines, consistent with increased membrane permeability and late-stage cell death processes. Taken together, these data suggest that cisplatin treatment alters cell death profiles and that these changes may contribute to the observed shift toward larger DNA fragments.

## 4. Discussion

Liquid biopsy approaches using ctDNA are increasingly being integrated into cancer management for applications like treatment monitoring. However, despite rapid clinical adoption, the biological mechanisms governing ctDNA release and fragmentation remain incompletely understood, particularly in the context of systemic therapy exposure. A better understanding of how chemotherapy influences ctDNA kinetics is critical for accurate interpretation of longitudinal liquid biopsy measurements. In this study, we used in vitro cancer cell models to investigate the biological impact of chemotherapy treatments on cfDNA release and fragmentation. We demonstrated that cfDNA release is not only proportional to viable tumor cell number but is also dynamically influenced by chemotherapy exposure and cellular sensitivity to treatment.

Clinical studies have shown that tumor burden is a key determinant of ctDNA abundance in patients [1,17,18]. While this relationship has most often been demonstrated in patient plasma, a previous study has also shown that cell line-specific cfDNA quantified from culture media strongly correlated with colony formation following irradiation in in vitro non-small cell lung cancer (NSCLC) models, and that cfDNA-derived survival curves were comparable to traditional clonogenic survival assays [19]. Consistent with these findings, we observed that viable cancer cell number correlated with cfDNA released into the culture medium across lung and esophageal cancer cell lines. This relationship held true using custom ddPCR assays for quantification of mutation-specific cfDNA, an approach analogous to ctDNA quantification in patient plasma [20]. Together, these findings support the expected relationship between tumor cell abundance and cfDNA release, while also establishing a controlled experimental framework in which the contribution of treatment exposure can be evaluated independently of total cell number.

We next demonstrated that chemotherapy generally increases per-cell cfDNA release in both lung and esophageal cancer models. Importantly, the two agents used—cisplatin, a platinum-based DNA-damaging agent, and 5-FU, an antimetabolite—have distinct mechanisms of action [21,22], yet both led to increased cfDNA release. These findings suggest that treatment-induced cancer cell death, irrespective of cell death mechanism, can increase cfDNA release into the extracellular environment. This is consistent with prior work from Rostami et al. showing that cfDNA release is governed by the cellular response to treatment, with cell death by apoptosis and necrosis both contributing to increased cfDNA release under different therapeutic contexts [3]. Overall, these data suggest that in vitro cfDNA levels reflect not only viable tumor cell number, but also treatment-induced biological processes.

Most importantly, we showed that there is a significant difference between the emission kinetics of cfDNA from chemosensitive and resistant cells after cisplatin treatment, which has significant implications for the interpretation of clinical liquid biopsy data. During longitudinal patient monitoring, rising ctDNA levels can be interpreted as evidence of increasing disease burden or resistance to therapies. However, our findings suggest that early increases in ctDNA following cytotoxic therapy may also reflect effective tumor cell killing, particularly when blood is sampled close to treatment administration. This may help explain clinical observations of transient ctDNA increases or early peaks following treatment initiation [4,5,6,23]. These ctDNA release patterns likely vary by treatment type, as therapies that induce inflammation and cell death may produce larger initial ctDNA spikes than primarily cytostatic therapies [24]. Moreover, recent clinical studies have demonstrated that early on-treatment ctDNA kinetics are associated with therapeutic response and long-term clinical outcomes [25]. For example, recent clinical studies in solid malignancies show that a rapid reduction or clearance of ctDNA within the first weeks of systemic therapy is associated with favorable treatment response and prolonged survival [26,27]. Together with our in vitro findings, these clinical observations support a time-dependent model of treatment-associated ctDNA kinetics, whereby early chemotherapy-induced tumor cell death may initially increase ctDNA release, followed by a decline in ctDNA as treatment-sensitive tumor cells are eliminated. In contrast, failure to clear ctDNA during subsequent treatment may reflect the persistence of treatment-resistant disease. Thus, clinicians and researchers interpreting rising ctDNA levels during platinum-based chemotherapy regimens should consider that, depending on the timing of sampling, such increases may paradoxically indicate effective tumor cell killing rather than disease progression. Future clinical studies incorporating high-frequency longitudinal sampling could help further characterize these ctDNA release and clearance kinetics in vivo.

Additionally, the distinct cfDNA release kinetics observed between chemosensitive and resistant cells also suggest that cfDNA could be used as a functional biomarker of therapeutic response during in vitro drug screens. In this context, cfDNA profiling may provide a multidimensional readout of tumor biology, capturing not only tumor-specific genetic alterations, but also chromatin organization, cell death processes, and dynamic responses to therapeutic exposure. Incorporating cfDNA measurements into preclinical drug-response studies may therefore provide complementary information to conventional viability or cytotoxicity assays and support more personalized approaches to therapy selection. Recent advances in precision oncology approaches have enabled the development of more physiologically relevant models of cancer, including patient-derived organoids and organ chip systems [28,29]. While our study focuses on 2D cell culture systems, these emerging models could be used in future studies to further expand and validate our findings in a more physiologically representative model. In fact, several studies have shown that cfDNA released from patient-derived organoids can recapitulate the biology of the primary tumor, including mutational profiles and nucleosomal footprints [30,31,32,33]. Together, these findings support the potential use of in vitro cfDNA profiling as a functional precision oncology tool for evaluating therapeutic response and advancing personalized treatment strategies.

Finally, given the emergence of fragmentomics as a promising field in liquid biopsy research, we sought to determine whether chemotherapy treatment has an impact on cfDNA fragmentation patterns. We observed a shift in the average length of cfDNA fragments following cisplatin treatment in both chemosensitive and resistant cells. Moreover, using annexin V/PI assays, we observed increased proportions of PI-positive and double-positive cell populations within each cell line following chemotherapy treatment, consistent with increased membrane permeability and late-stage cell death processes. These findings suggest that cfDNA fragment size may reflect the cellular processes contributing to DNA release, with the release of longer DNA fragments potentially associated with loss of membrane integrity and altered cell death processes, potentially including necrotic cell death. This has potential implications for the use of cfDNA fragmentation for treatment monitoring. Early changes in cfDNA fragmentation might help identify early response to therapy by providing insight into how tumor cells are dying following treatment. Importantly, these fragmentation patterns may vary according to the mechanism of action of the treatment. Different therapeutic agents can induce distinct forms of cell death, which may in turn produce different cfDNA fragmentation profiles. Systematic evaluation of fragmentation patterns across treatment classes could therefore help determine whether specific fragmentomic signatures are associated with particular mechanisms of drug-induced cellular responses and support clinical integration of fragmentomic assays. Controlled in vitro models are especially valuable for investigating these relationships because they allow for the assessment of treatment exposure, cellular sensitivity, and mechanism of cell death independently of physiological factors that influence cfDNA processing and clearance in patients.

**Figure 6:**
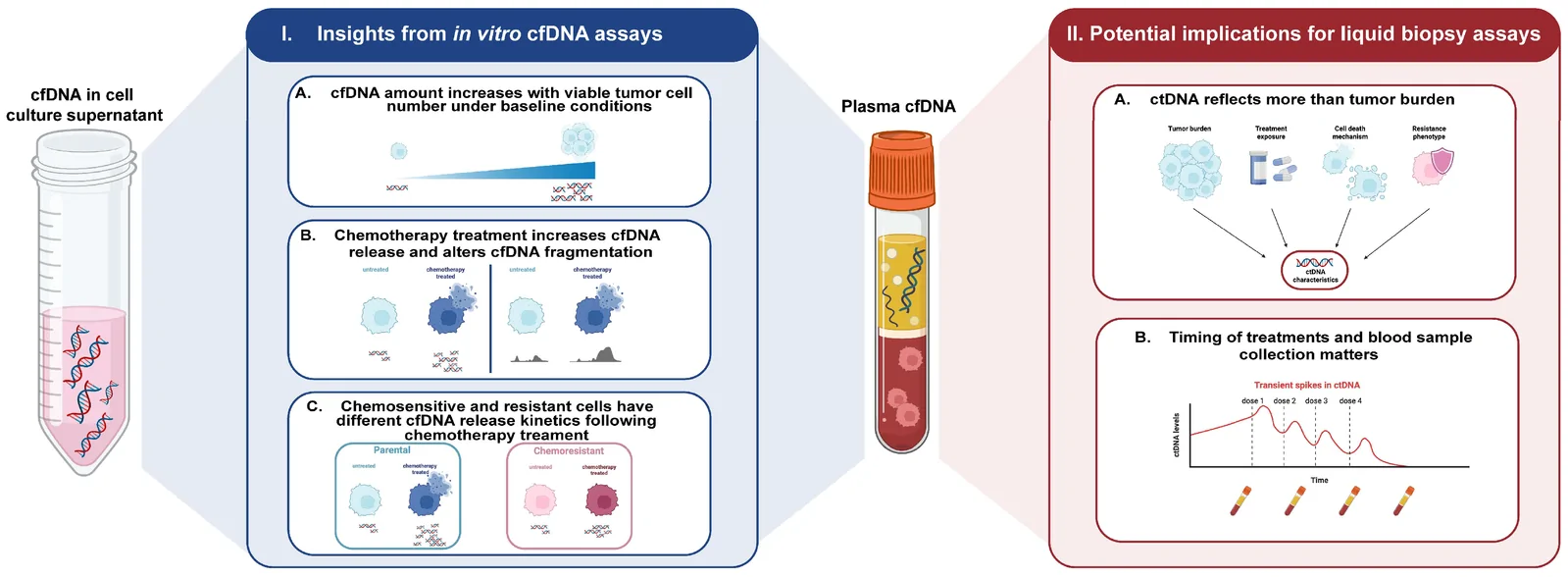
In vitro cfDNA release is a dynamic readout of tumor burden, treatment response, and resistance biology. Schematic summary of the biological findings from in vitro models of cfDNA release and their potential, hypothesis-generating implications for liquid biopsy assays.

Overall, combining cfDNA quantification analyses with fragmentomic profiling may provide a more comprehensive profile of therapeutic response. This approach might have applications in preclinical drug screening, since cfDNA characteristics could serve as complementary readouts of treatment efficacy and cell-death processes. Together, our findings suggest that ctDNA should be interpreted as a dynamic signal shaped by tumor burden, treatment timing, cell death mechanisms, and resistance phenotype, rather than as a direct measure of tumor mass alone. This model and its potential implications for liquid biopsy analyses are summarized in Figure 6.

This study had several limitations. All experiments were performed in vitro, and therefore the findings may not fully recapitulate the complexity of cfDNA kinetics and fragmentation in vivo. Our experimental system enabled the controlled investigation of tumor-derived DNA released directly into the surrounding culture medium; however, the abundance and detectability of ctDNA in clinical samples may also depend on the biofluid analyzed and its anatomical proximity to the tumor. Tumor-proximal biofluids may contain a greater relative abundance of ctDNA than peripheral blood, whereas ctDNA in the systemic circulation is present within a large background of cfDNA derived from non-malignant tissues and is additionally influenced by factors such as nuclease activity and clearance by immune cells and renal filtration [2,34]. Since the in vitro models used lacked the presence of physiological nucleases, the fragmentation profiles observed here likely reflect primary DNA release rather than the fully processed cfDNA typically seen in patient plasma samples. Furthermore, Annexin V/PI staining cannot definitively distinguish between late apoptosis, secondary necrosis, necroptosis, or other regulated cell death pathways, and therefore the specific mechanisms linking cell death to the observed fragmentation changes remain to be established. Additionally, cisplatin is known to induce DNA crosslinking and double-strand breaks, which may influence cfDNA fragment sizes [21]. The DNA extraction methodology may also differentially recover DNA fragments according to size and may therefore contribute to the observed fragment size distributions [35,36]. Also, while the cell-culture supernatants were processed by sequential centrifugation at 300× *g* and 1000× *g*, residual cellular debris cannot be completely excluded and may have also contributed to the larger DNA fragments detected.

The present study was focused on cytotoxic chemotherapy in solid tumor models. It would be informative to extend these analyses to chemotherapeutic agents with distinct mechanisms of action in order to determine whether the fragmentation changes observed here are treatment-specific. Immunotherapies, targeted therapies, and other newer treatment approaches may also induce distinct patterns of tumor-cell injury, immune-mediated killing, and cfDNA release [26,37]. Similarly, cfDNA kinetics in hematologic and lymphoproliferative malignancies may differ from those observed in solid tumors because of differences in disease distribution, tumor-cell turnover, treatment response, and the accessibility of ctDNA to the circulation [38]. Also, fixed chemotherapy concentrations were used throughout the cfDNA release experiments, and the dose-dependent effects of chemotherapy exposure on cfDNA release kinetics and fragmentation were not systematically evaluated. Future studies evaluating a range of drug concentrations may help determine how the magnitude of treatment-induced cytotoxicity influences cfDNA release and fragmentation patterns. Finally, the use of three independent biological replicates for most experiments may also limit statistical power, particularly for the interpretation of non-significant findings. Overall, future studies incorporating larger numbers of biological replicates, additional cancer models and therapy modalities, as well as in vivo validation will be important to further elucidate these mechanisms.

## 5. Conclusions

Overall, this study shows that in vitro cancer models provide a useful platform to investigate the fundamental biology of cfDNA release from cells. Our findings exemplify that cfDNA release and fragmentation are both dynamically influenced by chemotherapy treatment. Importantly, cfDNA release kinetics were found to reflect cellular response to chemotherapy treatment. These results may have implications for the timing of clinical liquid biopsy sampling and standardization of clinical ctDNA assays and provide a hypothesis-generating basis for future clinical studies.

## Figures and Tables

**Table 1:** Primer and probe sequences.

Cell Line	Mutation Targeted	Primer Sequences	Probe Sequences	Annealing Temperature (°C)
A549	*KRAS* G12S	F: CTGTATCGTCAAGGCACTCTT R: GTGTGACATGTTCTAATATAGTCACATT	WT:/5HEX/TGGAGC + T + G + GTGG/3IABkFQ/ MUT:/56-FAM/TTGGA + GC + T + A + G + TGG/3IABkFQ/	60
FLO-1	*TP53* C277F	F: GTAATCTACTGGGACGGAACAG R: CCCTTTCTTGCGGAGATTCT	WT: /5HEX/TT + GTGCC + T + G + TCCTG/3IABkFQ/ MUT:/56-FAM/TT + GT + GCC + T + T + T + CCTG/3IABkFQ/	62
OE19	*TP53* N310K	F: CACCTTTCCTTGCCTCTTTC R: TTCTCCATCCAGTGGTTTCT	WT:/5HEX/TGCC + C + A + A + CA + ACA/3IABkFQ/ MUT:/56-FAM/TGCC + C + AA + A + CA + ACA/3IABkFQ/	54

F: forward primer, R: reverse primer, WT: wild-type probe, MUT: mutant probe.

## Data Availability

The raw data supporting the conclusions of this article will be made available by the authors on request.

## Supplementary Materials

The following supporting information can be downloaded at: https://www.mdpi.com/article/10.3390/cells15171561/s1, Figure S1: Cells release large cfDNA amounts in the first 24 h after trypsinization. Cells were seeded and allowed to attach and grow for 24 h in culture media. Supernatant was then collected and cfDNA levels were quantified by (A) Qubit Fluorometer and (B) ddPCR. Data represents mean ± SD (*n* = 3 biological replicates); Figure S2: Preliminary assessment of cfDNA release from detached cells following chemotherapy treatment. Cisplatin Resistant OE19 cells were treated with 20 μg/mL of cisplatin for 48 h. The supernatant was collected and spun at 300× *g* for 5 min to pellet the detached cells. 100,000 and 500,000 detached cells were then plated for 6 h, supernatant was collected, and cfDNA was isolated. Total cfDNA levels quantified by Qubit Fluorometer are shown. Data represents mean ± SD (*n* = 2 biological replicates). This preliminary data supports the potential contribution of detached cells to extracellular cfDNA release; Figure S3: Background DNA detected in complete culture media and extraction blanks. Three independent 3 mL aliquots of complete F-12K, DMEM, and RPMI 1640 media containing 10% FBS, 1% penicillin–streptomycin, and 2 mM GlutaGro were incubated in empty T25 flasks for 24 h. Media controls were then processed using the same sequential centrifugation, DNA isolation, two-step elution, and Qubit dsDNA High Sensitivity quantification procedures as the experimental cell-culture supernatants. Three PBS extraction blanks were processed in parallel through the complete DNA extraction procedure using the same input volume. Data represent mean ± SD from three independent controls, each measured in technical triplicate; Figure S4: Optimization of mutation-specific ddPCR assays using annealing temperature gradients. Representative one-dimensional fluorescence amplitude plots are shown for assays targeting (A) KRAS G12S, (B) TP53 C277F, and (C) TP53 N310K. Each assay was evaluated using synthetic gBlock controls containing either the corresponding mutant sequence (left) or wild-type sequence (right). Mutant channel fluorescence is shown in the upper plots and wild-type channel fluorescence in the lower plots. Annealing temperatures from left to right for A-B: 64.0, 63.3, 61.7, 59.4, 56.7, 54.4, 52.9, and 52.0 °C. Annealing temperatures from left to right for C: 60.0, 59.4, 58.1, 56.2, 54.0, 52.1, 50.7, 50.0 °C. Mutant positive droplets are shown in blue, wild-type positive droplets in green, and negative droplets in grey. Gradients were used to identify assay-specific annealing temperatures that provided clear discrimination between positive and negative droplet populations while minimizing nonspecific signal in the alternate channel. The selected annealing temperature for each assay is reported in Table 1; Figure S5: Representative two-dimensional droplet plots for the mutation-specific ddPCR assays. Representative two-dimensional fluorescence amplitude plots are shown for assays targeting (A) KRAS G12S, (B) TP53 C277F, and (C) TP53 N310K at their optimized annealing temperatures. For each assay, plots are shown for the corresponding mutant synthetic gBlock positive control, no-template control (NTC), wild-type synthetic gBlock control, and a representative cfDNA sample from the relevant cell line: A549 for KRAS G12S, FLO-1 for TP53 C277F, and OE19 for TP53 N310K. Mutant positive droplets are shown in blue, wild-type positive droplets in green, and double-negative droplets in grey. The controls were used to confirm assay specificity, assess background fluorescence and nonspecific amplification, and establish droplet-classification thresholds for the experimental samples; Figure S6: Representative gating strategy for Annexin V/PI flow cytometry analysis. A representative sample from cisplatin resistant OE19 cells exposed to 0.002 mg/mL cisplatin for 72 h to illustrate the gating workflow. (A) Events were first gated by FSC-A and SSC-A to exclude debris. (B) Singlet cells were identified using FSC-A and FSC-H. (C) Annexin V and PI staining was assessed within the singlet population using quadrant gating to identify double-negative cells (Q4), Annexin V-positive/PI-negative cells (Q3), Annexin V/PI double-positive cells (Q2), and PI-positive/Annexin V-negative cells (Q1). The same gating strategy was applied across all experimental conditions; Figure S7: Cisplatin treatment leads to a shift in cfDNA fragment size. (A) Cell-free DNA ScreenTape electropherograms of extracellular DNA isolated from parental OE19 and cisplatin-resistant OE19 cells following 3 days of saline or cisplatin treatment. For each condition, traces from 3 independent biological replicates, each analyzed in 2 technical replicates, are shown (6 traces per condition). The prominent peaks at the lower size limit correspond to the internal TapeStation lower marker (LM). (B) Proportion of total cfDNA signal within the indicated fragment-size ranges. The 134–200 bp and 50–700 bp ranges were quantified using the Cell-free DNA ScreenTape assay. The 1–15 kb and >1 kb ranges were quantified using the Genomic DNA ScreenTape assay. Data are presented as mean ± SD from 3 independent biological replicates; Table S1: Unnormalized cfDNA concentrations and corresponding cell counts for the experiments presented in Figure 2 and Figure 4. Values are presented for individual biological replicates. Technical-replicate measurements, where applicable, were averaged to obtain one value per biological replicate. Total cell number was calculated as the sum of attached and detached cells. Normalized cfDNA values correspond to those presented in Figure 2 and Figure 4.

## Abbreviations

5-FU	5-fluorouracil
ANOVA	Analysis of variance
bp	Base pair
CCK-8	Cell Counting Kit-8
cfDNA	Cell-free DNA
ctDNA	Circulating tumor DNA
ddPCR	droplet digital polymerase chain reaction
DMEM	Dulbecco’s Modified Eagle Medium
DMSO	Dimethyl sulfoxide
EAC	Esophageal adenocarcinoma
FBS	Fetal bovine serum
FU	Fluorescence units
IC50	half-maximal inhibitory concentration
MRD	minimal residual disease
NaCl	Sodium chloride
NSCLC	Non-small cell lung cancer
NTC	No template control
PCR	polymerase chain reaction
PI	Propidium iodide
qPCR	quantitative polymerase chain reaction
SD	standard deviation
