# Supplementary material for: Cell-Free DNA Release Kinetics and Fragmentation Reflect Treatment Response and Resistance In Vitro
Source: Cells. 2026 Aug 28;15(17):1561. doi: 10.3390/cells15171561 (PMC13565368; doi:10.3390/cells15171561)
Supplement: Supplementary file 1 [file cells-15-01561-s001.zip › cells-4432287-supplementary.pdf]

Supplementary Materials

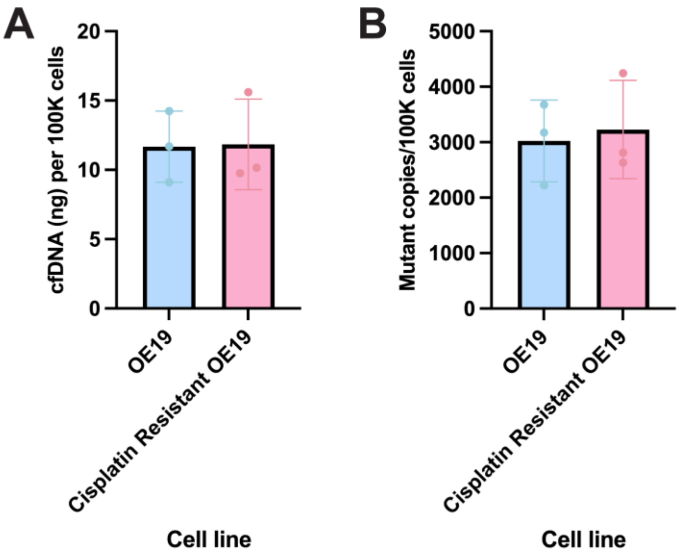

Supplementary Figure S1. Cells release large cfDNA amounts in the first 24 hours after trypsinization. Cells were seeded and allowed to attach and grow for 24 hours in culture media. Supernatant was then collected and cfDNA levels were quantified by **(A)** Qubit Fluorometer and **(B)** ddPCR. Data represents mean ± SD (*n*=3 biological replicates).

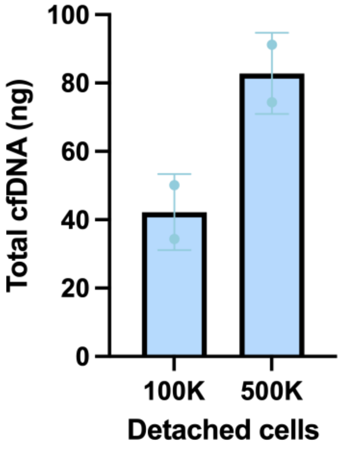

Supplementary Figure S2. Preliminary assessment of cfDNA release from detached cells following chemotherapy treatment. Cisplatin Resistant OE19 cells were treated with 20 μg/mL

of cisplatin for 48 hours. The supernatant was collected and spun at 300× *g* for 5 minutes to pellet the detached cells. 100K and 500K detached cells were then plated for 6 hours, supernatant was collected, and cfDNA was isolated. Total cfDNA levels quantified by Qubit Fluorometer are shown. Data represents mean ± SD (*n*=2 biological replicates). This preliminary data supports the potential contribution of detached cells to extracellular cfDNA release.

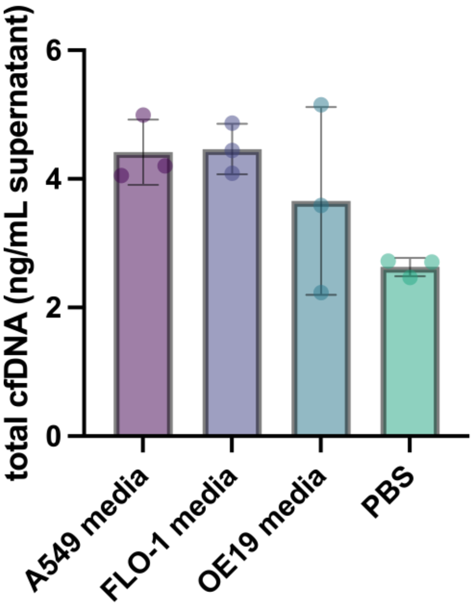

Supplementary Figure S3. Background DNA detected in complete culture media and extraction blanks. Three independent 3 mL aliquots of complete F-12K, DMEM, and RPMI 1640 media containing 10% FBS, 1% penicillin–streptomycin, and 2 mM GlutaGro were incubated in empty T25 flasks for 24 hours. Media controls were then processed using the same sequential centrifugation, DNA isolation, two-step elution, and Qubit dsDNA High Sensitivity quantification procedures as the experimental cell-culture supernatants. Three PBS extraction blanks were processed in parallel through the complete DNA extraction procedure using the same input volume. Data represent mean ± SD from three independent controls, each measured in technical triplicate.

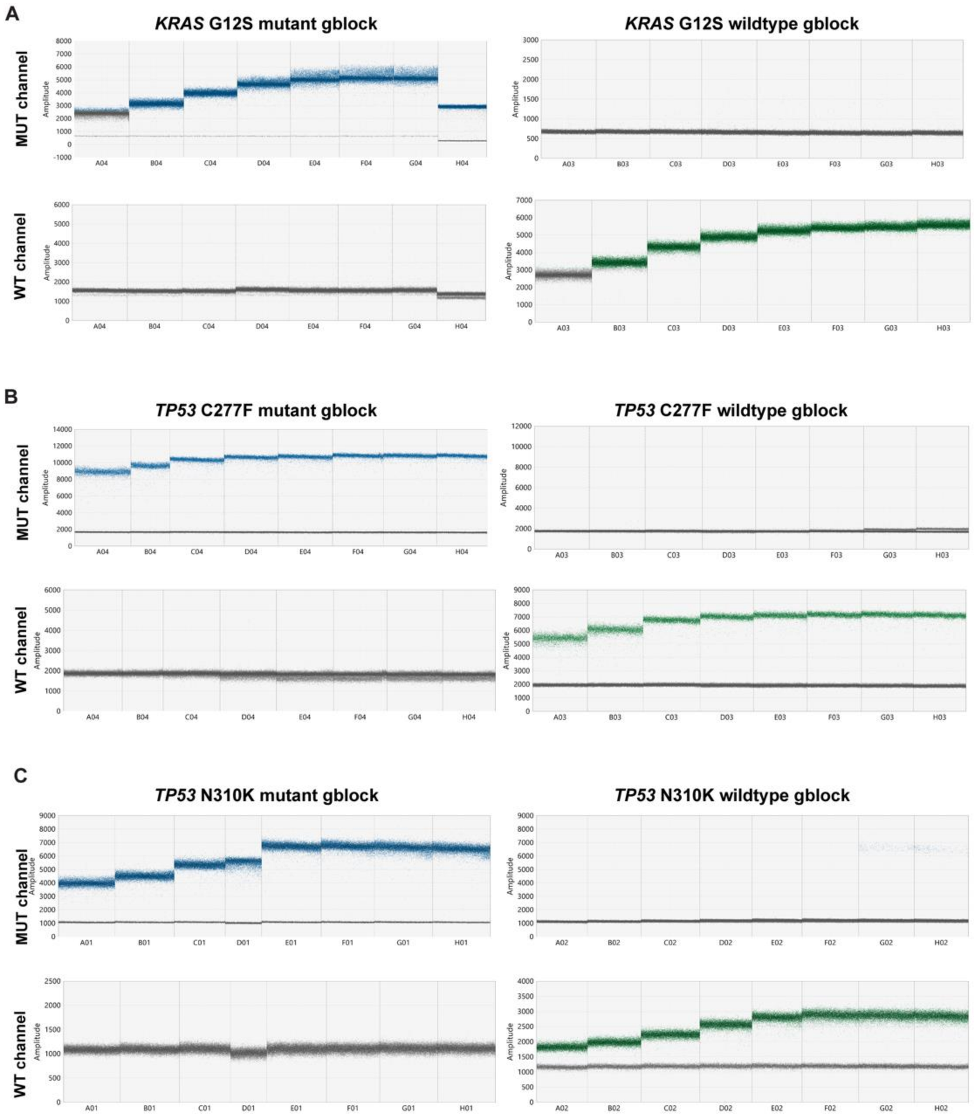

Supplementary Figure S4. Optimization of mutation-specific ddPCR assays using annealing temperature gradients. Representative one-dimensional fluorescence amplitude plots are shown for assays targeting **(A)** *KRAS* G12S, **(B)** *TP53* C277F, and **(C)** *TP53* N310K. Each assay was evaluated using synthetic gBlock controls containing either the corresponding mutant sequence (left) or wild-type sequence (right). Mutant channel fluorescence is shown in the upper plots and wild-type channel fluorescence in the lower plots. Annealing temperatures from left to right for A-B: 64.0, 63.3, 61.7, 59.4, 56.7, 54.4, 52.9, and 52.0 °C. Annealing temperatures from left to right for C: 60.0, 59.4, 58.1, 56.2, 54.0, 52.1, 50.7, 50.0 °C. Mutant positive droplets are shown in blue,

wild-type positive droplets in green, and negative droplets in grey. Gradients were used to identify assay-specific annealing temperatures that provided clear discrimination between positive and negative droplet populations while minimizing nonspecific signal in the alternate channel. The selected annealing temperature for each assay is reported in **Table 1**.

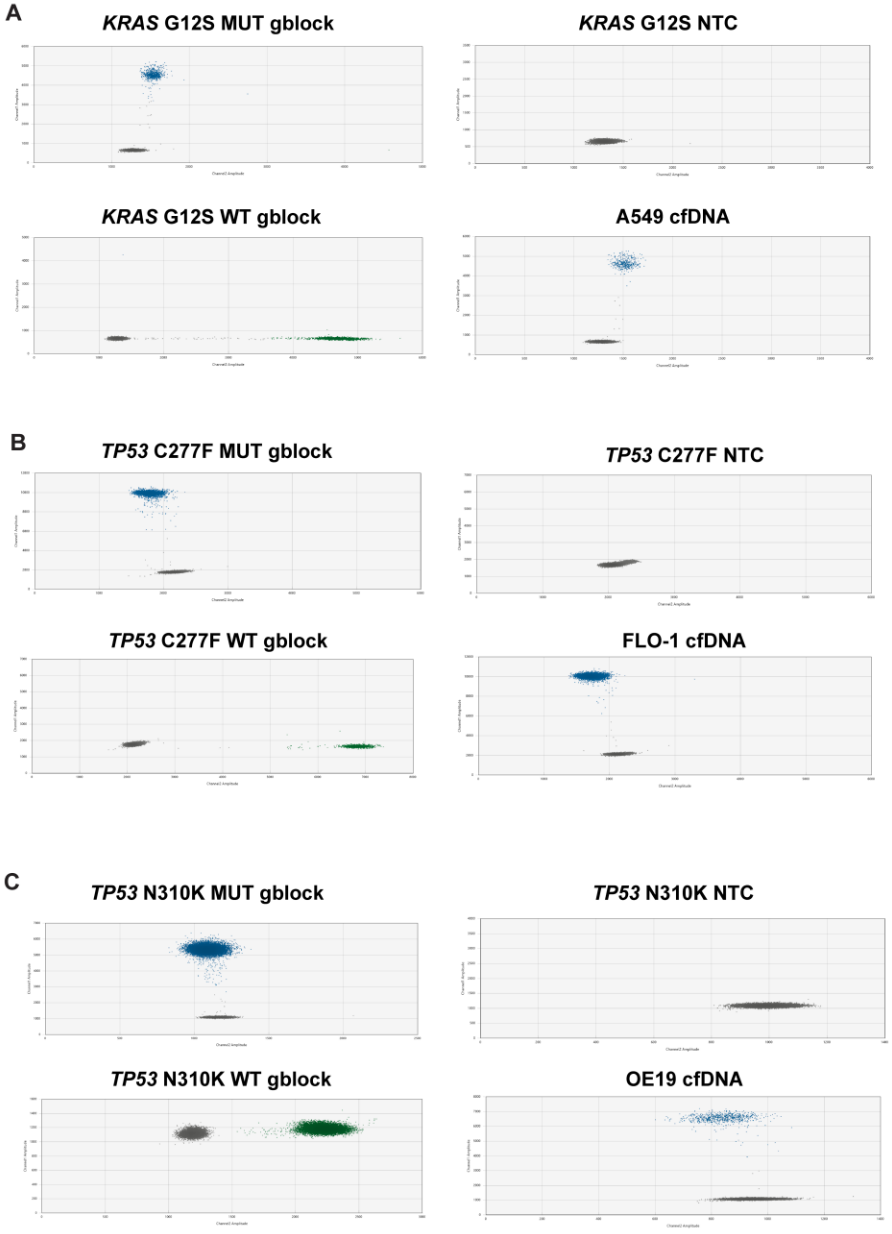

Supplementary Figure S5. Representative two-dimensional droplet plots for the mutation-specific ddPCR assays. Representative two-dimensional fluorescence amplitude plots are shown

for assays targeting **(A)** *KRAS* G12S, **(B)** *TP53* C277F, and **(C)** *TP53* N310K at their optimized annealing temperatures. For each assay, plots are shown for the corresponding mutant synthetic gBlock positive control, no-template control (NTC), wild-type synthetic gBlock control, and a representative cfDNA sample from the relevant cell line: A549 for *KRAS* G12S, FLO-1 for *TP53* C277F, and OE19 for *TP53* N310K. Mutant positive droplets are shown in blue, wild-type positive droplets in green, and double-negative droplets in grey. The controls were used to confirm assay specificity, assess background fluorescence and nonspecific amplification, and establish droplet-classification thresholds for the experimental samples.

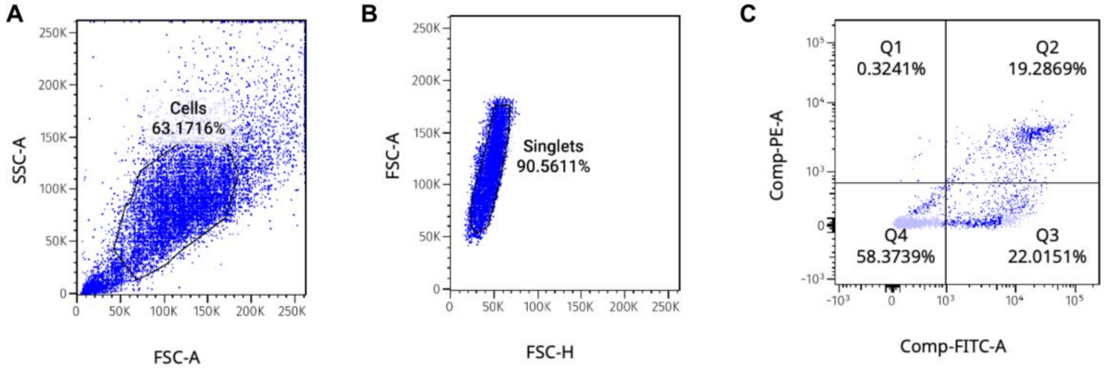

Supplementary Figure S6. Representative gating strategy for Annexin V/PI flow cytometry analysis. A representative sample from cisplatin resistant OE19 cells exposed to 0.002 mg/ml cisplatin for 72 hours to illustrate the gating workflow. **(A)** Events were first gated by FSC-A and SSC-A to exclude debris. **(B)** Singlet cells were identified using FSC-A and FSC-H. **(C)** Annexin V and PI staining was assessed within the singlet population using quadrant gating to identify double-negative cells (Q4), Annexin V-positive/PI-negative cells (Q3), Annexin V/PI double-positive cells (Q2), and PI-positive/Annexin V-negative cells (Q1). The same gating strategy was applied across all experimental conditions.

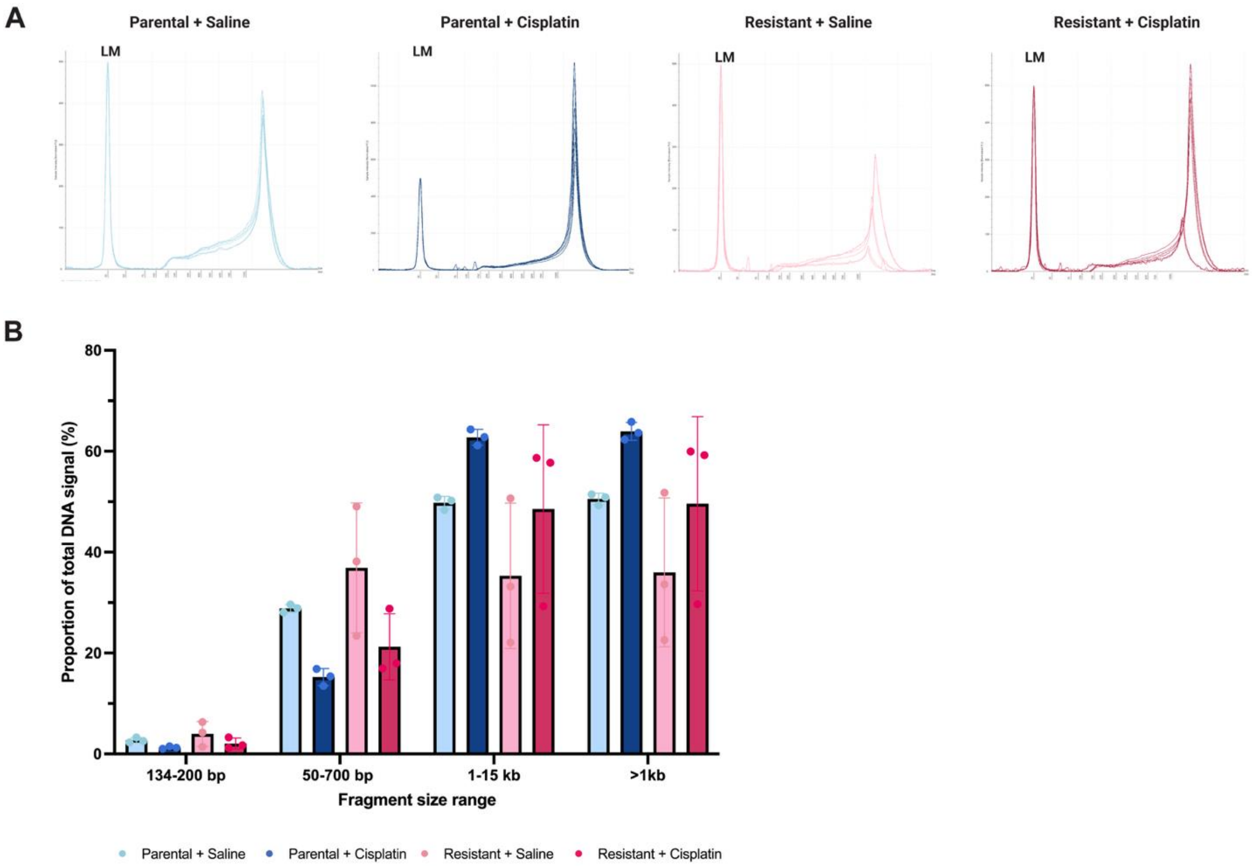

Supplementary Figure S7. Cisplatin treatment leads to a shift in cfDNA fragment size. (A) Cell-free DNA ScreenTape electropherograms of extracellular DNA isolated from parental OE19 and cisplatin-resistant OE19 cells following 3 days of saline or cisplatin treatment. For each condition, traces from 3 independent biological replicates, each analyzed in 2 technical replicates are shown (6 traces per condition). The prominent peaks at the lower size limit corresponds to the internal TapeStation lower marker (LM). **(B)** Proportion of total cfDNA signal within the indicated fragment-size ranges. The 134-200 bp and 50-700 bp ranges were quantified using the Cell-free DNA ScreenTape assay. The 1-15 kb and >1 kb ranges were quantified using the Genomic DNA ScreenTape assay. Data are presented as mean ± SD from 3 independent biological replicates.
